# WHF Position Statement on COVID Vaccination

**DOI:** 10.5334/gh.1027

**Published:** 2021-04-27

**Authors:** F. Thienemann, G. Chakafana, D. Piñeiro, F. J. Pinto, P. Perel, K. Singh, J.-L. Eiselé, D. Prabhakaran, K. Sliwa

**Affiliations:** 1 General Medicine and Global Health (GMGH), Cape Heart Institute, Faculty of Health Sciences, University of Cape Town, Cape Town, ZA; 2 Department of Medicine, Faculty of Health Sciences, Groote Schuur Hospital, University of Cape Town, Cape Town, ZA; 3 Department of Internal Medicine, University Hospital Zurich and University of Zurich, Zurich, CH; 4 Cape Heart Institute, Faculty of Health Sciences, University of Cape Town, Cape Town, ZA; 5 Universidad de Buenos Aires, AR; 6 Santa Maria University Hospital, CAML, CCUL, Faculdade de Medicina da Universidade de Lisboa, Lisbon, Portugal, CT; 7 Department of Non-communicable Disease Epidemiology, London School of Hygiene & Tropical Medicine, World Heart Federation, CH; 8 Public Health Foundation of India, Gurugram, Haryana, IN; 9 Centre for Chronic Disease Control, New Delhi, Public Health Foundation India, Gurugram, Haryana, India World Heart Federation, London School of Hygiene & Tropical Medicine, GB; 10 Division of Cardiology, Department of Medicine, Faculty of Health Sciences, Groote Schuur Hospital, Cape Town, ZA

**Keywords:** COVID vaccination, cardiovascular disease, global health

## Abstract

The current COVID-19 pandemic has challenged health systems and communities globally. As such, several countries have embarked on national COVID-19 vaccination programmes in order to curb spread of the disease. However, at present, there isn’t yet enough dosages to enable vaccination of the general population. Different vaccine prioritization strategies are thus being implemented in different communities in order to permit for a systematic vaccination of individuals. Here, on behalf of the World Heart Federation, we emphasize the need for individuals with Cardiovascular disease to be prioritized in national vaccine prioritization programmes as these are high risk individuals.

## Summary of COVID-19 vaccine landscape

Coronavirus disease 2019 (COVID-19) caused by Severe Acute Respiratory Syndrome Coronavirus 2 (SARS-CoV-2) has caused more than 2.5 million deaths worldwide within the first year of the pandemic [1]. SARS-CoV-2 is a single stranded, positive-sense, RNA virus that encodes many open reading frames (ORFs) [2]. The viral ORFs are responsible for the synthesis of several accessory and structural proteins such as the transmembrane spike (S) glycoprotein and the replicase enzyme. Recognition and attachment of the S protein to human epithelial cells lined with ACE2 receptors are important events in the hallmark of COVID-19 pathophysiology. As such, the S protein has become a leading target in the development of novel vaccines and neutralising antibodies against SARS-CoV-2. The urgent development of COVID-19 vaccines has been accelerated by the increased demands for intensive care facilities and exhaustion of the medical facilities as well as the economic consequences of successive lockdowns that most countries, in different forms, have followed. Vaccines possess great potential in mitigating COVID-19 as findings suggest that the action of neutralizing antibodies is crucial for viral clearance [3].

**Figure 1:**
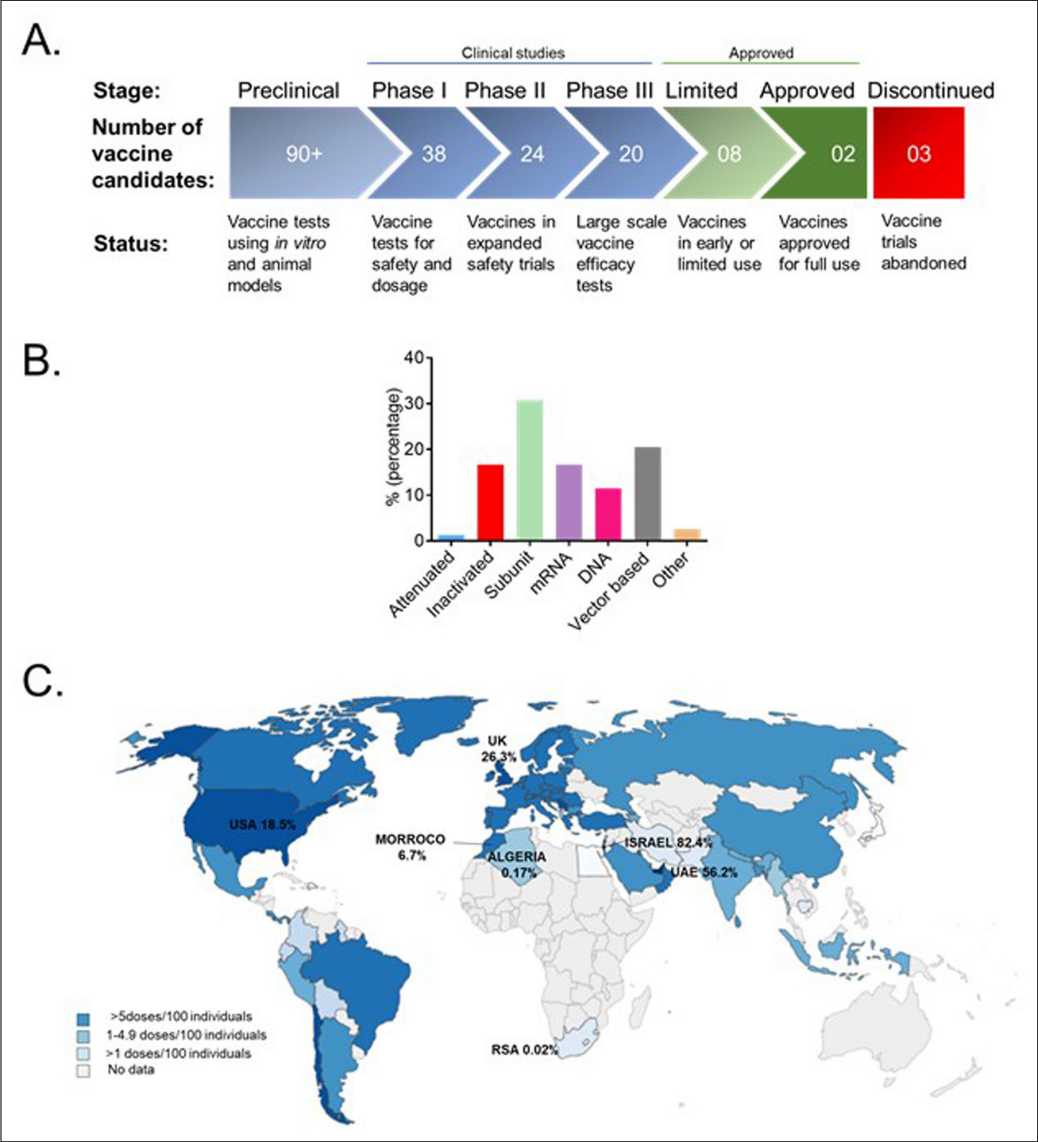
**Landscape of COVID-19 vaccines. (A)** Several vaccines are in the production pipeline at several development stages. **(B)** Although different approaches are being employed in the development of COVID-19 vaccines, many of the vaccines currently undergoing clinical trials are subunit and vector-based vaccines. **(C)** The global distribution of COVID-19 vaccine recipients as at 22 February 2021. Data source: Covid-19 Vaccine Tracker Updates: The Latest – The New York Times (nytimes.com); COVID19 Vaccine Tracker (trackvaccines.org); https://ourworldindata.org/covid-vaccinations.

Various approaches are under investigation to develop an effective and safe COVID-19 vaccine. Tremendous efforts from the scientific community, governments, and private sector have to date resulted in hundreds of vaccine-related projects aimed at mitigating the spread of the deadly virus (https://covid19.trackvaccines.org). At present, over 80 vaccine candidates are undergoing clinical testing in more than 220 clinical trials (Figure 1). Several approaches are being utilized in the development of efficacious COVID-19 vaccines to elicit an immune response upon exposure to non-virulent forms of the viral antigens, preventing future infection from the virus. Potential COVID-19 vaccines currently under development can be classified into the following categories: live/attenuated, inactivated, protein subunit, nucleic acid-based (mRNA or DNA-based) and vector-based (Table 1). With such diversity in the vaccine design approaches, it is not surprising that distinct population groups may respond differently to the various vaccine types. Therefore, the merits and demerits of each vaccine type relative to target recipient groups is an important consideration for vaccination programs. Much as some strategies possess great promise, there are also challenges associated with other vaccine design approaches. For instance, whereas protein subunit vaccines, coupled with adjuvants, are generally thought to induce a robust immune response [4], they require longer production time which demand complex downstream purification steps. In the case of a pandemic where emergency medical interventions are required as a matter of urgency, this approach may be less suitable. On the other hand, mRNA vaccines are much faster and relatively easier to produce, although their ultra-low temperature storage requirement is less convenient in low resource settings (Table 1). It is however important to have a variety of vaccine types as we are already seeing in many countries since this will allow the vaccines to complement each other in developing herd immunity globally.

**Table 1:** COVID-19 vaccine types, mechanisms, and features.

Vaccine type	Mechanism of action	Advantages	Disadvantages

**Live attenuated**	Produce by growing the virus in unfavourable conditions or by generating a genetically weakened version of the virus.	(1) Relatively higher efficacy than inactivated vaccines. (2) Immune response is directed against many SARS-CoV-2 antigens.	(1) Production and handling are associated with major biosafety risks. (2) May be unsuitable for use in some age groups.
**Inactivated**	SARS-CoV-2 is inactivated by exploiting different chemical techniques to produce a vaccine.	(1) More stable than live attenuated vaccines. (2) Immune response is directed against many SARS-CoV-2 antigens.	(1) Short duration of immune memory which demands inoculation of higher vaccine doses. (2) May result in hypersensitivity.
**Subunit**	The S protein or its fragments are produced by rDNA technology to make a vaccine.	(1) Elicit robust immune response, when combined with adjuvants.	(1) Require stringent downstream purification steps which are often expensive.
**DNA-based**	DNA plasmids are used to induce cells to produce the S protein, thus activating an immune response.	(1) Great flexibility for manipulation of the coded antigen. (2) Quick to produce. (3) High antibody titres.	(1) Specialized and complex delivery (electroporation). (2) Repeated doses may cause toxicity. (3) Relatively lower immune responses.
**mRNA**	mRNA vaccines temporarily induce cells to produce the antigen protein encoded.	(1) Low production costs. (2) Quick to produce.	(1) Vaccine preparations must be kept at ultralow temperatures.
**Vector**	DNA coding for the S protein is conveyed into cells by viral vectors. By inserting the DNA in a virus, it is possible to exploit the virus’s great ability to infect and deliver the mRNA into the human cells.	(1) Candidate vaccines may induce a mucosal immunity capable of neutralizing the virus, thus inhibiting its ability to enter the human body.	(1) Possibility of presenting varied immune responses.

Other less common technological approaches such as the use of probiotics, heat-inactivated plasma and dendritic cells are also being explored in COVID-19 vaccine development [5]. The orally administered probiotic, *Bifidobacterium*, is currently undergoing Phase I clinical trials [6]. Trials are also being conducted on dendritic cells that are engineered to express the SARS-CoV-2 antigen [7], thus initiating an immune response. Interestingly, it seems the majority of COVID-19 vaccine candidates presently undergoing clinical trials are S protein subunit-based vaccines (Figure 1; Table 2). Subunit vaccines coupled with adjuvants are generally known to elicit a robust immune response in several disease models [8]. The emerging increase in SARS-CoV-2 mutations is a key concern which arises from the sole reliance on S proteinbased vaccines. SARS-CoV-2 variants in South Africa, the United Kingdom (UK) and Brazil have recently been described [9]. The South African 501Y.V2 variant is characterised by three mutations; K417N (a lysine to asparagine substitution at amino acid position 417 in the S protein), E484K (a glutamic acid to lysine substitution at amino acid position 484 in the S protein) and N501Y (an asparagine to tyrosine substitution at amino acid position 501) which all occur in the S protein [10]. However, the effect of these mutations on S protein-based vaccines is yet to be fully determined. Live attenuated and inactivated vaccines composed of multiple antigens may therefore be more efficient options as they can elicit an immune response not only directed towards the S protein. Nonetheless, long-term immunity and safety of inactivated vaccines requires further confirmation as previous studies with inactivated SARS-CoV vaccines have resulted in lung eosinophilia [11].

**Table 2:** List of all 12 vaccines currently approved by at least one country (Date: 28 February 2021).

Developer	Name	Type	Countries in use	Immunogenic features	Efficacy

FBRI/Novavax	EpiVacCorona	Protein	01	High levels of S-specific neutralizing antibodies.	Not available
Pfizer/BioNTech	BNT162b2	mRNA	65	2 repeated doses (28 days apart) which induce elevated concentrations of neutralizing antibody titers. Also induce CD4^+^ and CD8^+^ T cells responses.	95%
Moderna	mRNA-1273	mRNA	40	2 repeated doses (28 days apart) which induce neutralizing antibodies and CD4^+^ and CD8^+^ T cell responses.	94.1%
Janssen (Johnson & Johnson)	Ad26.COV2.S	Vector	1	Single dose vaccine inducing neutralizing antibodies.	85%
CanSino	Ad5-nCoV	Vector	3	Strong immune response with single delivery but impeded due to pre-existing immunity.	65.7%
Gamaleya	Sputnik V	Vector	38	Induces high neutralizing antibody titers. Also induces CD4+ and CD8+ T cells responses.	91.4%
Oxford/Astra Zeneca	AZD1222	Vector	56	Strong immune response and high neutralizing antibodies with single injection (Low pre-existing immunity).	62%
Serum Institute of India	Covishield	Vector	13	Oxford-AstraZeneca vaccine is being manufactured locally by the Serum Institute of India	Similar to Oxford/Astra Zeneca
Bharat Biotech	Covaxin	Inactivated	2	N/A	81%
Sinopharm (Wuhan)	Vero Cells	Inactivated	2	Enhanced induction of neutralizing antibodies and enhanced immunogenicity.	79%
Sinovac	CoronaVac	Inactivated	12	Elevated induction of neutralizing antibodies and enhanced immunogenicity.	50-91%*
Sinopharm (Beijing)	BBIBP-CorV	Inactivated	16	Safe and high antibody titers.	79-86%*

* *variations in results obtained in different trials.* *Data retrieved from the COVID19 vaccine tracker of scientists of McGill University, Canada, accessed on 28 February 2021 (https://covid19.trackvaccines.org/vaccines)*.

At the time of this article submission, more than 15 vaccines have been approved for emergency use in different countries (https://covid19.trackvaccines.org) and this is changing rapidly (Table 2). Notably, many of the currently approved vaccines are vector-based and inactivated vaccines (Table 2). Most recombinant COVID-19 viral vector vaccines act as immunogens that are engineered to facilitate the expression of a variety of antigens. It is however worth noting that two mRNA vaccines, from Pfizer and Moderna, have been already approved in many countries and are thus being used for most vaccination programs globally (Figure 1). These vaccines are characterised by the mRNA sequence of the S protein, carried by lipid microvesicles (liposomes). A key advantage of mRNA-based vaccines is that they facilitate the transient expression of antigenic peptides thus evoking robust major histocompatibility complex-I (MHC-I) presentation and cytotoxic T lymphocytes (CTL) responses. Data from clinical trials on both vaccine regimens revealed that the safety was overall reassuring as no unexpected patterns of concern were identified [12]. Reported side effects upon vaccination ranged from fatigue, myalgia, arthralgia, and headache [13]. Both vaccines have been shown to possess over 94% efficacy (Table 2) and have thus been opted into many COVID-19 vaccination programs worldwide.

### Importance of COVID-19 vaccine for people with cardiovascular disease

**Figure 2:**
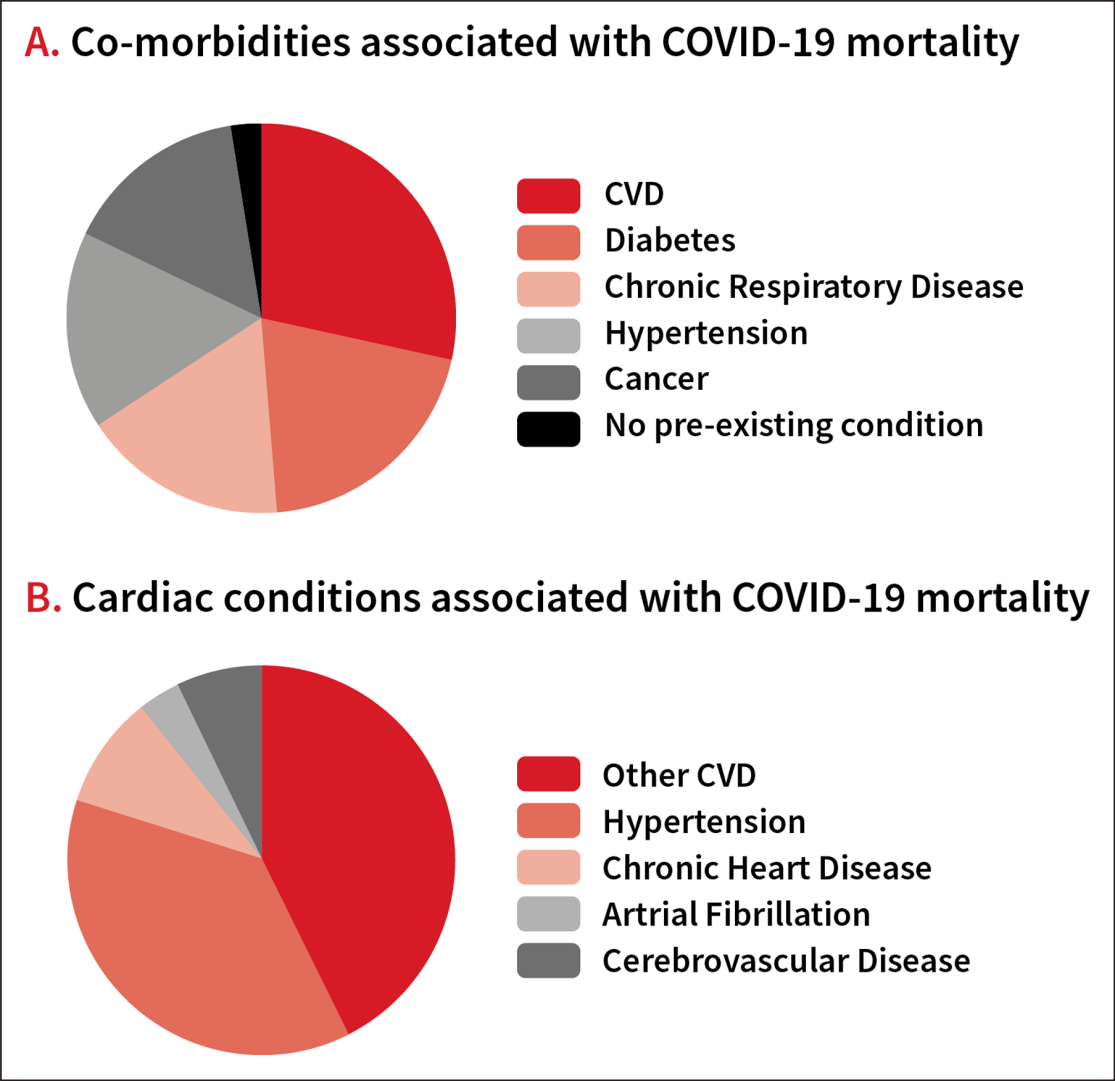
**Distribution of CVD risk factors in COVID-19 mortality. (A)** CVD and hypertension are responsible for most of COVID-19 related mortalities. **(B)** Hypertension is a major contributor to COVID-19 mortality. Data sourced from study by Li et al., 2020 [19].

Numerous studies have looked at the association of cardiovascular disease (CVD) and its risk factors with COVID-19 severity. For example in a Chinese study, the COVID-19 mortality rate was 2.3% in individuals without any pre-existing medical conditions, and was elevated to 6.0% for patients with hypertension, 7.3% for patients with diabetes, and 10.5% for patience with CVD [14]. Other studies have showed that both the susceptibility and outcomes of COVID-19 strongly correlate with CVD [15,16,17,18]. Indeed, evidence from several cohort studies has indicated that CVD accounts for approximately between 15–36% of total COVID-19 mortalities (Figure 2) [19,20]. Studies have shown that CVD patients present a threefold risk for progression towards serious disease, as evidence by the higher number of patients either requiring mechanical ventilation (22% vs. 8%), ICU (intensive care unit) (13% vs. 4%) admission or dying (17% vs. 5%) [19]. In addition, CVD patients develop complications such as acute respiratory distress syndrome, malignant arrhythmias, and acute kidney injury in the course of disease [19]. Results from a multicentre cohort study of 191 patients with COVID-19, also indicated that out of 33 patients with acute cardiac injury, 32 died [20]. CVD is thus an important COVID-19 risk factor requiring for early intervention and mitigation and therefore individuals with CVD should be prioritized as a high risk group for vaccination. This is particularly crucial because more than 400 million individuals across the globe have CVD [21]. However, the disproportionate burden of CVD in low- and middle-income countries, where vaccine dissemination is still limited is worrisome.

**Figure 3:**
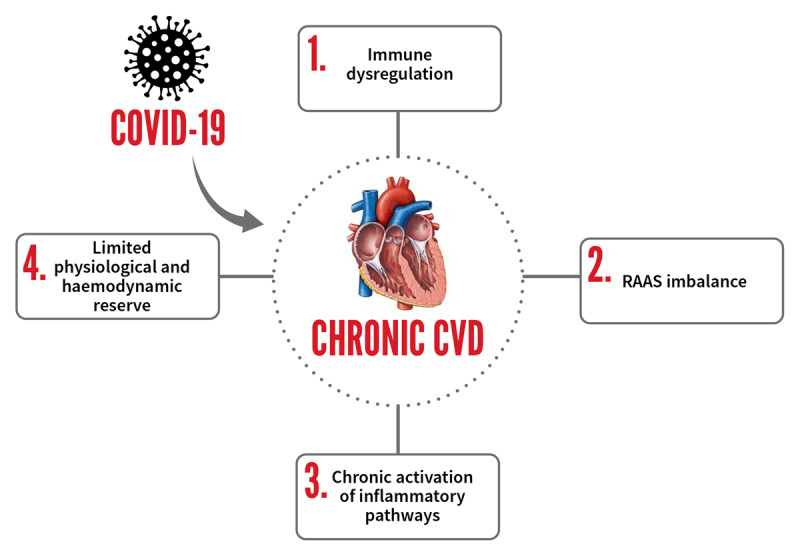
Outcomes of COVID-19 infection in CVD patients.

Although, SARS-CoV-2 generally results in mild disease in approximately 80% of the cases, some individuals progress to a severe respiratory illness characterized by hyperinflammatory syndrome, multiorgan dysfunction, and death [22]. There are several mechanisms via which individuals with CVD and hypertension present a greater risk of severe COVID-19 clinical presentation or mortality (Figure 3). The first mechanism involves a dysregulation of ACE2 receptors in vascular endothelial cells of CVD patients [22,23]. Following initial recognition of ACE2 receptors by the S protein, the metallopeptidase domain protein (ADAM17) expression is upregulated to facilitate cleavage of ACE2 from the cell membrane [24]. This results in a loss of ACE2-mediated protection against the effects of the renin angiotensin aldosterone system (RAAS) which in turn promotes vascular contraction and endothelial injury. In addition, epithelial injury also causes an up-regulation of tissue factor expression and imbalance of fibrinolysis system [25]. Evidence from previous studies has indeed shown that ACE2 expression on the cell surface is reduced after SARS-CoV-2 infection [26]. ACE2 reduction induces an exaggerated release of several pro-inflammatory cytokines into the circulation, thus inducing a cytokine storm [27]. Together these events may trigger critical illness and inflammation which may further aggravate pre-existing cardiovascular disorders including heart failure, ischemia, and arrhythmias [23]. Notably, an upsurge in inflammatory biomarkers in COVID-19 patients with CVD has been reported, indicating that inflammatory cell necrosis promoted inflammatory response leading to myocardial damage, or fulminant myocarditis [28,29]. In this manner, CVD may worsen outcomes in COVID-19 patients.

CVD may also result in further complications in COVID-19 patients due to the association of chronic CVD with a myriad of other risk factors such as obesity and diabetes which are additional COVID-19 risk factors. In fact, the American Heart Association reports that approximately 68% of people with diabetes (who are over 65 years) die from heart disease. A combination of CVD and diabetes therefore puts individuals at further risk of COVID-19 related mortality. Furthermore, CVD and diabetes may result in immune dysregulation which predisposes individuals at risk of a more severe COVID-19 clinical manifestation [30]. Evidence from previous work has highlighted a relationship between CVD and immune dysregulation [31,32] and this association may trigger worse outcomes in COVID-19 patients. It is also worth noting that CVD are linked to chronic activation of inflammatory pathways. Therefore, it can also be speculated that since SARS-CoV-2 infection generally elicits an inflammatory response, a synergistic inflammatory cascade may possibly be triggered in CVD patients, resulting in COVID-19 related complications and multi organ failure.

It is thought that SARS-CoV-2 can directly replicate inside the myocardium resulting in primary myocardial injury which may further aggravate pre-existing myocardial injury [33,34]. It is unknown if the effects of direct viral replication in the heart may be amplified in patients with chronic CVD, especially in those with coronary artery disease. From previous evidence, viral infection was shown to cause endothelial dysfunction leading to accelerated atherogenesis and induction of coronary plaque instability [35,36]. These cardiac complications thus present a greater risk of mortality in CVD patients. It is also plausible that CVD patients are at high risk from SARS-CoV-2 infection due to their limited physiological and haemodynamic reserve under oxidative stress. Heart failure patients generally have reduced cardiac output, which is further burdened by reduced pulmonary function during COVID-19. This increased pressure may further stress CVD patients resulting in adverse events from COVID-19. Increased oxidative stress and elevated blood pressure can in turn damage vascular endothelium causing hypercoagulability in COVID-19 patients with CVD [37].

Recent reports also indicate that COVID-19 patients with CVD frequently developed complications including acute liver injury and acute renal injury during hospitalization [19]. Patients with CVD were also reported to exhibit poor compensatory ability of cardiac function and inflammatory cytokine storm, which exacerbated microcirculation ischemia and hypoxia of liver cells and further aggravated liver function injury [19]. Taken together, these findings suggest that individuals with CVD present a high risk as CVD is usually associated with worse COVID-19 outcomes.

### COVID-19 vaccine access – Global equitable and fair distribution

Since late 2020 several regulators have approved different COVID-19 vaccines including the European Medicines Agency (EMA), the U.S. Food and Drug Administration (FDA) and the U.K. Medicines and Healthcare products Regulatory Agency (MHRA). The three more widely approved vaccines that have so far been approved by these stringent authorities are Pfizer/BioNTech, Oxford/AstraZeneca, and Moderna. The World Health Organization Strategic Advisory Group of Experts on Immunization (SAGE) has recently issued interim recommendations for use of the Oxford/AstraZeneca COVID-19 vaccine [38].

Intense discussions around the world arose on 1) global equitable and fair access and 2) allocation of COVID-19 vaccines. South Africa is the first country in Africa that has started a vaccination program on 18^th^ February 2021. At the time of this article submission, Israel has by far the most widely vaccinated population, with approximately 43 doses per 100 individuals (Figure 1). Although most of the European population is undergoing an intensive vaccination program, so far only between 1–11% of the population has been vaccinated. In contrast, vaccination in low- and middle-income countries is much slower with most of Africa is yet to be vaccinated against COVID-19. Initial vaccination programs are prioritising different populations, but in general most strategies include frontline health and social care workers, individuals over 65 years, and people with existing chronic conditions who are considered to be at high risk of developing severe COVID-19. It is important to emphasize that individuals with CVD should be considered among the latter and should be included as a priority in such vaccination programs.

Back in April 2020, the *Access to COVID-19 Tools (ACT) Accelerator* was initiated to address those issues. ACT Accelerator is a unique global collaboration led by the World Health Organization to support the development and equitable distribution of COVID-19 tests, treatments and vaccines. *COVID-19 Vaccines Global Access (COVAX)* is one of three pillars of the ACT Accelerator and offers a global solution to the COVID-19 pandemic. COVAX is a platform to accelerate the development and manufacture of COVID-19 vaccines, and ‘to ensure that people in all corners of the world will get access to COVID-19 vaccines once they are available, regardless of their wealth [39].’ It is also very important to implement strategies that will allow for massive and fast vaccination as soon as vaccines become available. The example of the successful massive vaccination program in Israel supports this concept. Close to 90% of people aged 60 and older in the country have received their first dose of Pfizer/BioNTech two-dose prime-boost vaccine so far. There was a 41% drop in confirmed COVID-19 infections in that age group, and a 31% drop in hospitalizations from mid-January to early February [40]. In addition, recent data published online showed that vaccination reduced the viral load by 1.6x to 20x in individuals who were positive for SARS-CoV-2. This estimate might improve after more individuals receive the second dose. The overall direction is clear: COVID-19 vaccination with the Pfizer/BioNTech vaccine can 1) protect individual from severe COVID-19 disease, and 2) can also reduce transmission and halt the spread of COVID-19 and potentially end the pandemic [41].

## Vaccine Prioritization

Initial vaccination programs are prioritising different populations, but in general, most include the groups identified as Tier 1 by the World Health Organization including frontline health and social care workers, people over 65 years, and people under the age of 65 years who have underlying conditions that put them at a higher risk of death.

### The importance of COVID-19 vaccination for people with cardiovascular disease

Among the latter a very important group is people with CVD, as mentioned this population is at high risk for severe disease and should be considered as priority individuals for vaccination. By reducing the likelihood of infection in this population, not only will there be a direct impact and decrease in mortality, but also a reduction of patients with the need of intensive care facilities including mechanical ventilation. This will help to reduce the current pressure on the healthcare systems, which is one of the main issues many countries are facing and directly related with the total number of infected people as well as the number of high-risk patients being infected, such as those with CVD.

### The importance of COVID-19 vaccination for healthcare workers

COVID-19 vaccine allocation - often referred to as vaccine prioritization - sparked again highly emotional discussions when healthcare workers were not considered in the first phase of vaccination campaigns. Vaccine rollout plans were developed at national levels on how to distribute COVID-19 vaccines in light of potentially limited supply during the early rollout phase. While efforts were often focussing on elderly people and people at high risk for severe disease, healthcare workers and other frontline workers at high daily risk of exposure to SARS-CoV-2 are not always considered for vaccination during the first phase. As a result, many healthcare professionals around the globe raised their voices to be heard [42]. In South Africa, the petition ‘We need a COVID-19 vaccine plan now!’ called on the Department of Health to start ‘vaccinating frontline health workers, followed by those most at risk including elderly people and people with comorbidities,’ a motion that was supported by Professor Salim Abdool Karim, chairperson of the COVID-19 Ministerial Advisory Committee of South Africa.

### Healthcare workers should be prioritized to ensure the continuous care of populations

Many healthcare workers around the world get infected at the workplace and die of COVID-19. According to the Centers for Disease Control and Prevention (CDC) COVID data Tracker, more than 400,000 healthcare personnel cases occurred in the US since the beginning of the epidemic until end of February 2021 claiming 1,371 lives [43]. Healthcare workers infected with COVID-19 need to self-isolate, and consequently, many healthcare workers with contact to infected healthcare workers need to be quarantined. Thus, entire healthcare services and hospitals, for example the Humboldt Hospital in Berlin [43], were shut down - often for weeks - and could not serve the general population. Healthcare workers can also spread COVID-19 to patients and residence of long-term facilities. Those individuals often have underlying conditions that increase the risk of severe COVID-19 disease. Also, healthcare workers can spread the virus to colleagues at work leading to outbreaks amongst staff [44]. Vaccinating healthcare personnel will therefore help to prevent intrahospital transmission of COVID-19 and to reduce the nosocomial spread of COVID-19 in health services.

Healthcare workers should be immunised first and immediately to protect healthcare capacity and for hospitals to maintain safe and operational levels to serve the population continuously, especially in the light of emerging SARS-CoV-2 variants that are more infective and lead to increased pressure on healthcare systems globally.

In conclusion, COVID-19 pandemic represents a major threat to humankind in many respects, including, but not limited, to health and economic related issues. The unprecedented fast development of safe and effective vaccines as well as the fast–track emergency use and approval of COVID vaccines by the regulatory authorities brought the hope to curtail this threat. To achieve this, vaccination against COVID-19 should be seen as a global priority involving all countries. The success against SARS-CoV-2 is dependent of how much ‘global’ reach will be achieved in a concerted and timely manner. The key factors for success will be based on: equity between and within countries on the distribution of vaccines, massive administration and prioritization of access based on objective criteria (health care workers, to ensure healthcare is uninterrupted, and high-risk groups such as people aged over 65 years and people with chronic conditions such as CVD, to reduce severe complications and subsequent pressure on health systems), education of the populations to improve trust, and acceptance of COVID-19 vaccine, and, last but not least, ensure the implementation of mechanisms for mass production of vaccines at a global level.
